# Split-Dose Polyethylene Glycol Is Superior to Single Dose for Colonoscopy Preparation: Results of a Randomized Controlled Trial

**DOI:** 10.1155/2016/3181459

**Published:** 2016-04-13

**Authors:** Rachid Mohamed, Robert J. Hilsden, Catherine Dube, Alaa Rostom

**Affiliations:** ^1^Division of Gastroenterology, Faculty of Medicine, University of Calgary, Calgary, AB, Canada; ^2^Departments of Medicine and Community Health Sciences, University of Calgary, Calgary, AB, Canada; ^3^Forzani & MacPhail Colon Cancer Screening Centre, Alberta Health Services, Calgary, AB, Canada; ^4^Division of Gastroenterology, Faculty of Medicine, University of Ottawa, Ottawa, ON, Canada

## Abstract

*Background*. The efficacy of colonoscopy in detecting abnormalities within the colon is highly dependent on the adequacy of the bowel preparation. The objective of this study was to compare the efficacy, safety, and tolerability of PEG lavage and split-dose PEG lavage with specific emphasis on the cleanliness of the right colon.* Methods*. The study was a prospective, randomized, two-arm, controlled trial of 237 patients. Patients between the age of 50 and 75 years were referred to an outpatient university screening clinic for colonoscopy. Patients were allocated to receive either a single 4 L PEG lavage or a split-dose PEG lavage.* Results*. Overall, the bowel preparation was superior in the split-dose group compared with the single-dose group (mean Ottawa score 3.50 ± 2.89 versus 5.96 ± 3.53; *P* < 0.05) and resulted in less overall fluid in the colon. This effect was observed across all segments of the colon assessed.* Conclusions*. The current study supports use of a split-dose PEG lavage over a single large volume lavage for superior bowel cleanliness, which may improve polyp detection. This trial is registered with ClinicalTrials.gov identifier NCT01610856.

## 1. Introduction

The efficacy of colonoscopy in detecting abnormalities within the colon is highly dependent on the adequacy of the bowel preparation. Despite advances in bowel preparation, the process remains difficult for patients to tolerate and complete and ultimately, if inadequate, can result in missed lesions [1]. With published literature on missed polyps and carcinomas at colonoscopy [2–4], optimal visualization of the colonic mucosa becomes critically important. Furthermore, some evidence suggests that colonoscopy may not be protective against right-sided colonic lesions [5, 6]. Subtle flat lesions with a predilection for the proximal colon, particularly those with serrated histology, are becoming increasingly significant as potential factors for the lack of protection of colonoscopy proximally and stress the importance of adequate preparations [7–10].

*Background.* Polyethylene glycol (PEG) is a balanced electrolyte lavage rather than an osmotic agent; therefore its use as a bowel-cleansing preparation is typically associated with fewer fluid shifts and electrolyte abnormalities compared with low volume osmotic agents [11]. It has been widely used for colonoscopy preparation on its own [12–16] or in conjunction with other agents [17–20]. One disadvantage of the traditional 4 L PEG bowel preparation is a reduction in the quality of cleanliness with afternoon procedures [21], which has driven further research into timing and dosing of PEG depending on timing of the procedure [22–26]. In our previous study, the 4 L PEG preparation was somewhat inferior to sodium phosphate preparations (now removed from the market) because it left a large amount of residual fluid in the colon [27]. However, the use of a split dose of PEG (2 L the day before the procedure and 2 L the morning of the procedure) may alleviate some of the shortcomings of a single 4 L preparation, particularly for afternoon procedures [28].

The objective of this study was to compare the efficacy and tolerability of single- and split-dose PEG lavage, with specific emphasis on the cleanliness of the right colon and the influence of endoscopy time.

## 2. Materials and Methods

All patients between the age of 50 and 75 years referred to the Forzani & MacPhail Colon Cancer Screening Centre in Calgary, Alberta, Canada, for colonoscopy in 2010 were considered for inclusion. During preassessments at the clinic, patients were asked to participate in the study by a nurse clinician. Those not interested in participating simply received the Centre's standard bowel preparation protocol.

Patients with acute coronary syndrome, congestive heart failure, unstable angina, known or suspected renal failure, ascites, megacolon, known or suspected bowel obstruction, or other comorbidities that may prevent colonoscopy were excluded. Patients were also excluded if they previously had a partial or subtotal colectomy or if the colonoscopy was warranted for the evaluation of diarrhea.

Enrollment of participants was performed by a study coordinator. A computer was used to generate a randomizations table with blocks of 8. Allocation concealment was maintained through the use of consecutively numbered sealed envelopes. The allocation ratio was 1 : 1. Colonoscopists and investigators were blinded to the allocation groups.

Patients were allocated to one of two groups: (1) 4 L PEG the day before the procedure (starting at noon for AM procedures and 6:00 PM for PM procedures) or (2) 4 L of PEG in a split dose (procedure before 10 AM: 2 L at 12 noon and 2 L at 8 PM the day before the procedure; procedure after 10 AM: 2 L of PEG at 8 PM the day before the procedure, and 2 L of PEG 5 hours before the scheduled procedure time on the day of the procedure).

A study assistant assigned patients to their group and instructed them on the proper use of their assigned bowel preparation method. Patients were instructed to start a low residue diet 4 days prior to the colonoscopy. A table of acceptable foods (white bread, white pasta, dairy, eggs, chicken, beef, pork, fish, cooked or steamed vegetables, and canned fruits) and foods to avoid (whole grain, brown or wild rice, oatmeal, raw fruits and berries, any food containing nuts, seeds, and popcorn) was included in the patient handout. Patients were instructed to take a light breakfast on the morning before the procedure, followed by clear fluids thereafter. Patients were given a tolerability questionnaire that was modified from a previously reported questionnaire [27], to be completed once their bowel preparation was finished and before coming to the hospital for the colonoscopy. Patient concerns or questions regarding the preparation were directed to the assistant as opposed to their gastroenterologist, so as to avoid unblinding the gastroenterologist.

### 2.1. Outcomes

The previously validated Ottawa Bowel Preparation scale [29] was used to assess the quality of bowel cleanliness. Each of the right, mid, and rectosigmoid colons was rated on a 5-point scale (0–4). In addition, overall colonic fluid was assessed with a 3-point rating (0–2), resulting in an overall score range of 0 to 14. An excellent preparation with little fluid would have a score of 0 to 1 and a good preparation would have a score of 2 to 4. An adequate bowel preparation would have a score of 5 or lower and a completely unprepared colon would have a score of 11 to 14, depending on the amount of colonic fluid.

Colonoscopy was performed in a standard fashion and endoscopists rated the bowel preparation quality during the procedure before any attempts to improve visualization and recorded the result on a separate standardized form.

Secondary outcomes included a tolerability questionnaire, as well as patient and investigator reported adverse events.

### 2.2. Statistical Analyses

Descriptive statistics were used for baseline characteristics. The Ottawa Bowel Preparation scale produces data that are approximately normally distributed. Two-group ANOVA was used to assess for the presence of group differences in the preparation scores while accounting for procedure time (AM versus PM). The proportion of adequate bowel preparation in each group was compared with a chi square statistic.

The secondary endpoints of tolerability were assessed using the Mann-Whitney *U* test.

### 2.3. Sample Size Calculation

In our previous study using the Ottawa Bowel Preparation scale, an observed effect size of 1.0 was seen [27]. For a two-group ANOVA, a total sample size of 190 patients would be required for a two-tailed alpha at 0.05 and an 80% power. Based on experience from our previous study, an additional 20% of patients (*n* = 38) were added to the total sample size to account for early withdrawals and incomplete colonoscopies.

## 3. Results

In total, 249 patients were enrolled in the study: 125 in the split-dose group and 124 in the single-dose group; 237 completed the study, 115 patients in the single-dose group and 122 patients in the split-dose group. Discontinuations were due to scheduling conflicts. No adverse events causing discontinuations were reported.  Table 1 shows the baseline characteristics of the two groups. There were no significant differences between groups. In the split-dose group, 41% underwent afternoon colonoscopies compared with 40% in the single-dose group.

**Figure 1:**
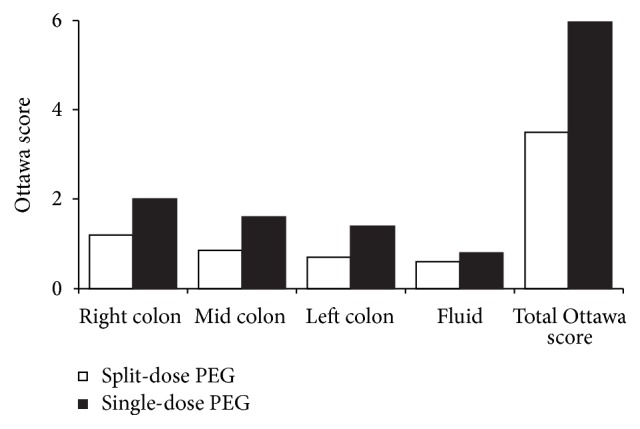
Ottawa Bowel Prep scores for split-dose and single-dose PEG lavage groups by colon segment and overall. Lower scores are better.

**Figure 2:**
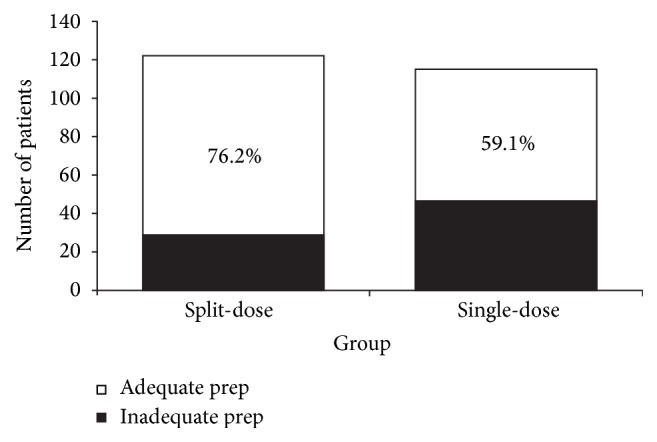
Proportion of patients with adequate bowel preparation (Ottawa Bowel Prep score <6) for split-dose and single-dose PEG lavage groups.

The differences in Ottawa score between the two groups are illustrated in Figure 1. In total, the bowel preparation was superior in the split-dose group compared with the single-dose group (mean Ottawa score: 3.50 ± 2.89 versus 5.96 ± 3.53; *P* < 0.05). Split-dose PEG resulted in a lower, and therefore better, mean Ottawa score across all segments of the colon assessed as well as less overall fluid in the colon. This reached statistical significance for the total, right, and left colon. Furthermore, the split-dose group had a higher proportion of patients with adequate bowel preparation (Ottawa score < 6) than the single-dose group (76.2% versus 59.1%; *P* < 0.001; Figure 2).

Split preparations for morning procedures were performed only for those procedures after 10 AM, as earlier procedures were not feasible to have patients take an early morning dose of their preparation. In procedures performed in the morning, the split-dose PEG lavage resulted in a lower total Ottawa score compared with the single-dose lavage (mean total Ottawa score: 4.31 ± 3.13 versus 5.51 ± 3.61; *P* = 0.035). The Ottawa score in the right colon was significantly better in the split-dose group than in the single-dose group (1.51 ± 1.06 versus 1.88 ± 1.08; *P* = 0.042). The differences in Ottawa score between the two groups for morning procedures are illustrated in Table 2.

**Figure 3:**
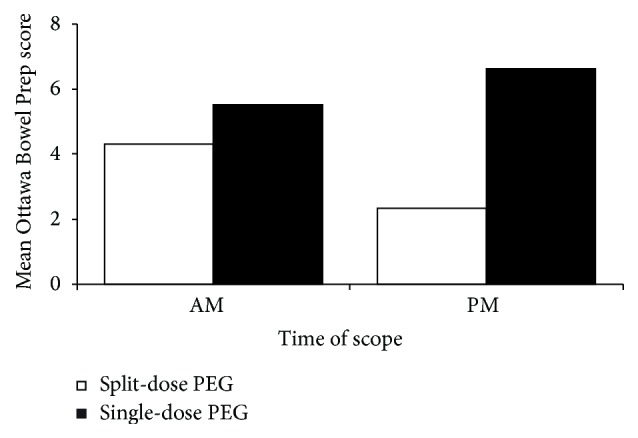
Bowel preparation quality for AM (earlier than 10:00 AM) and PM (after 10:00 AM) procedures. All single-dose patients took their bowel preparation (4 L PEG) the day before the colonoscopy starting at noon for AM procedures and 6:00 PM for PM procedures. Split-dose patients took the second dose the evening before colonoscopy for AM procedures, or 5 hours before the colonoscopy time for PM procedures.

The effect of the split-dose lavage compared with the single-dose lavage is amplified in the afternoon procedures. The total Ottawa score was far superior in the split-dose arm (2.34 ± 2.02 versus 6.63 ± 3.33, *P* < 0.005) as were the independent assessments of the different colonic regions (right colon 0.82 versus 2.22; mid colon 0.58 versus 1.91; left colon 0.40 versus 1.52; all *P* < 0.005). The differences in total Ottawa score with the split-dose and single-dose PEG lavage for both morning and afternoon procedures are illustrated in Figure 3.

Both preparations were generally well tolerated and completed as directed by the majority of participants (90% in split-dose group versus 85% in single-dose group; *P* = NS). Questions regarding tolerability of the lavage preparations are shown in Table 3. More patients who took the single 4 L lavage would rather have had a different preparation method compared with those who took the split dosing. The mean number of adverse events experienced during the lavage was higher in the single-dose group compared with the split-dose group (2.60 versus 2.01; *P* = 0.05). Adverse event data are provided in Table 4.

## 4. Discussion

Administration of a PEG lavage in two 2 L doses is superior to administration in a single 4 L dose for screening colonoscopy in an outpatient population. The result is an overall better cleanliness in all regions of the colon as well as less residual fluid. While this holds true for procedures performed throughout the day, the effect is amplified when there is a shorter interval between the last dose of PEG and the procedure, as was the case in the afternoon procedures where the second PEG dose was given in the morning of the day of the procedure. In this situation, the difference in total Ottawa score between the split-dose and single-dose preparations was the greatest. While there was still a significant difference in morning procedures where the last PEG dose was given the evening before, the effect was less dramatic. This begs the question as to whether patients with morning procedures should be asked to take the second PEG dose 4-5 hours before the procedure rather than the evening before, at least in the setting of colorectal cancer screening. Clearly, this puts a significant added burden on patients with scheduled morning procedures.

There are a variety of reasons as to why a split-dose administration of PEG is superior to a single-dose administration. The second dose of the split preparation is given closer to the procedure time than is the single dose. The result is likely a better removal of fluid and debris that may have accumulated in the hours since the first dose of the PEG lavage [22, 30, 31]. The single dose of PEG is almost always given the night before the procedure, leaving several hours since the laxative was administered for fluid, bile, and debris to accumulate. Therefore the bowel becomes less clean over time. Matro and colleagues have recently shown equivalency for morning only and a split-dose PEG preparation (PM/AM) for afternoon procedures, suggesting that the earlier dose may not even be required [25]. Another study found that maximal cleanliness of the bowel preparation was achieved when the colonoscopies were performed within 8 hours of the last laxative dose [30]. This notion is supported in our study by the superior bowel preparations in the afternoon procedures. The effect was less pronounced in the morning procedures likely because of the increased time between the second dose (evening before) and the colonoscopy time. In our study, afternoon procedure cases received the second dose of PEG 5 hours priorly.

A better bowel preparation has been associated with higher cecal intubation rates and higher polyp detection rates [30]. In a study by Marmo et al., a split-dose PEG lavage outperformed a single-dose PEG lavage for colonoscopy. The improved bowel preparations were associated with a 5-fold increase in cecal intubation and a 2-fold increase in adenoma detection [30]. The superiority of the split-dose lavage in the right colon shown in this study and others [32] facilitates better identification of the serrated adenomas, which tend to be flat and more difficult to detect. Polyp and adenoma detection rates are typically described in similar trials now; however, as this study was conducted in 2010, detection rates for each treatment group are not available.

The split-dose PEG lavage is well tolerated and preferred by patients over the single-dose lavage [33]. A survey by Unger and colleagues found that 85% of patients would be willing to wake up in the middle of the night to take a second dose of bowel lavage for a morning procedure [34]. Furthermore, they found a 78% compliance rate with the split-dose protocol in those with morning scheduled colonoscopies, which are the most difficult to prepare for as the second dose of bowel lavage is needed at a very early morning hour. For later scheduled procedures, benefits of the split-dose protocol include less interruption as the patient can often rest for an extended period of time after the first laxative dose compared with the larger volume single-dose method [32]. In our study, the split-dose lavage was well tolerated and associated with fewer side effects than the single-dose lavage. Our results are also supportive of a recent systematic review which found that a 4 L split-dose lavage was superior to other bowel preparations [28].

This study was conducted in an outpatient colon cancer screening center. These results, therefore, may not necessarily apply to hospitalized patients, or those being evaluated for symptoms. Additionally, the centre employs a two-stage precolonoscopy assessment visit that includes a group learning session followed by an individualized nurse assessment where the bowel preparation strategy is again discussed. While both study groups attended this visit, the overall compliance of these patients may be greater than in a typical outpatient setting. A further limitation of the current study is that the timing of bowel preparation in the single-dose group may be earlier than what is routinely used in clinical practice.

The optimal preparation for outpatient colonoscopy continues to be an important area of interest and study. The current study supports use of a split-dose PEG lavage over a single large volume lavage. Ideal circumstances would have all patients take a second dose of bowel lavage approximately 5 hours before their scheduled procedure. The benefits are improved bowel cleanliness, which translates into improved polyp detection without increased adverse effects or patient dissatisfaction. This may justify the use of split-dose PEG lavage as the bowel preparation of choice for outpatient colonoscopies.

## Figures and Tables

**Table 1:** Baseline characteristics of the patients.

	Split-dose PEG	Single-dose PEG
Number enrolled	125	124
Drop-outs	3	9
Number analyzed	122	115
Mean age	55.5	53.5
Male	80 (64%)	66 (53%)

PEG: polyethylene glycol.

**Table 2:** Mean Ottawa scores parameters for morning procedures.

	Split-dose PEG	Single-dose PEG	*P* value
Right colon	1.51	1.88	0.042
Mid colon	1.19	1.43	0.212
Left colon	0.99	1.39	0.031
Total fluid	0.61	0.80	0.090
Total Ottawa	4.31	5.51	0.035

Ottawa score by colon segment and total Ottawa score for the entire colon. Lower numbers are better. PEG: polyethylene glycol.

**Table 3:** Questionnaire: patient opinion regarding the assigned bowel preparation.

Question	Split-dose PEG	Single-dose PEG	*P* value
% that completed the lavage as directed	90.0	85.0	0.28
% that would rather take a different lavage	42.4	52.4	0.045
% that would refuse the same lavage	8.0	10.0	0.25

PEG: polyethylene glycol.

**Table 4:** Adverse events (group symptom score).

	Split-dose PEG	Single-dose PEG	*P* value
Nausea	0.49	0.59	NS
Vomiting	0.22	0.05	0.013
Abdominal pain	0.30	0.59	0.001
Bloating	0.68	1.03	0.004
Chest pain	0.02	0.06	NS
Dizziness	0.25	0.25	NS
Shortness of breath	0.04	0.03	NS
Ankle swelling	0.01	0.01	NS
Total	2.01	2.60	0.051

Proportion of patients self-reporting bowel preparation related adverse symptoms or signs. There were no discontinuations due to adverse events. NS: not significant; PEG: polyethylene glycol.
